# Impairment of Diverse Patient-Reported Outcome Measures in Patients with Takayasu Arteritis: A Cohort Study

**DOI:** 10.31138/mjr.050625.qtr

**Published:** 2026-03-01

**Authors:** Darpan R Thakare, Upendra Rathore, Kritika Singh, Tooba Qamar, Vikas Agarwal, Manas Ranjan Behera, Neeraj Jain, Manish Ora, Durga Prasanna Misra

**Affiliations:** 1 Department of Clinical Immunology and Rheumatology, Sanjay Gandhi Postgraduate Institute of Medical Sciences (SGPGIMS), Lucknow, India;; 2 Department of Nephrology, Sanjay Gandhi Postgraduate Institute of Medical Sciences (SGPGIMS), Lucknow, India;; 3 Department of Radiodiagnosis, Sanjay Gandhi Postgraduate Institute of Medical Sciences (SGPGIMS), Lucknow, India;; 4 Department of Nuclear Medicine, Sanjay Gandhi Postgraduate Institute of Medical Sciences (SGPGIMS), Lucknow, India

**Keywords:** Takayasu arteritis, patient reported outcome measures, quality of life, anxiety, depression, physical activity

## Abstract

**Aim:**

To comprehensively evaluate patient-reported outcome measures (PROMs) and fibromyalgia in patients with Takayasu arteritis (TAK) compared with healthy controls.

**Methods:**

Quality of life (QoL) using EuroQol-5D-3L (EQ-5D), disability using Health Assessment Questionnaire (HAQ), physical activity – Metabolic Equivalents of Task (METs) using International Physical Activity Questionnaire – Short Form (IPAQ-SF), work impairment using Work Productivity and Activity Impairment – General Health (WPAIGH), anxiety using Generalised Anxiety Disorder seven items (GAD-7), depression using Patient Health Questionnaire nine items (PHQ-9), fatigue using Multidisciplinary Fatigue Inventory (MFI) and Functional Assessment of Chronic Illness Therapy (FACIT) scales and fibromyalgia were assessed in patients with TAK. Comparisons were performed between PROMs in patients with TAK and age- and sex-similar healthy controls, TAK with active or inactive disease by physician global assessment (adjusted for Large Vessel Vasculitis Index of Damage scores), or the same patients with TAK over time.

**Results:**

Eighty-four patients with TAK (mean age 35.61 years, 61 females) were compared with 61 healthy controls (mean age 33.62 years, 45 females). Patients with TAK had worse QoL, disability, activity impairment due to health, anxiety, depression and fatigue than controls (p<0.01); similar proportions had fibromyalgia. After adjustment for damage scores, patients with active TAK had worse impairment of physical activity due to health and fatigue. PROMs remained stable on follow-up in the patients with TAK (mean follow-up interval 7.53 months).

**Conclusion:**

QoL, disability, anxiety, depression, and fatigue are impaired in patients with TAK and remain stable over time. Fatigue is associated with active TAK.

## INTRODUCTION

Takayasu arteritis (TAK) is a rare large vessel vasculitis. TAK more commonly affects female patients. TAK might begin insidiously with a long asymptomatic period before incidental detection during routine physical examination or when ischemic complications develop. TAK is a difficult disease to treat. No drug has been shown to be effective in patients with TAK when compared with a placebo in a randomised controlled trial (RCT).^1–3^ In part, this is related to challenges in the assessment of activity and damage in patients with TAK.^3,4^

Increasingly, the importance of patient-reported outcome measures (PROMs) is being recognised in patients with rheumatic diseases including systemic vasculitis.^5,6^ PROMs refer to standardised assessment tools to assess the impact of disease on patients reported by the patients themselves. These include the assessment of various domains such as quality of life, disability, anxiety, depression, fatigue, physical activity and work productivity.^4,7,8^ Recent recommendations from the American College of Rheumatology (ACR) and European Alliance of Associations for Rheumatology (EULAR) for the management of rheumatic diseases highlight the importance of PROMs. Increasingly, PROMs are relevant to the approval of new drugs by regulatory agencies such as the United States Food and Drug Administration.^7^

The literature on PROMs in TAK is limited owing to its rarity. A recent systematic review with meta-analysis identified twenty-one studies (all but one observational) which had assessed PROMs in patients with TAK. This review had identified worse quality of life, disability, anxiety and depression in patients with TAK than in healthy controls. Active TAK was associated with poorer quality of life, greater work impairment and depression than inactive TAK.^8^ Fibromyalgia is another patient-reported and physician-assessed measure which is associated with worse PROMs in the context of different rheumatic diseases and has been scarcely evaluated in patients with TAK.^9^ A comprehensive assessment of patient-reported outcomes and fibromyalgia in patients with TAK is lacking. Therefore, this study aimed to evaluate the gamut of PROMs in patients with TAK compared with healthy control subjects, assess their stability on follow-up, and explore the relationship of PROMs with disease activity and damage in TAK.

## MATERIALS AND METHODS

Prevalent adult patients with TAK (age ≥ 18 years) were recruited from an ongoing cohort at a tertiary care centre in North India after seeking written informed consent (document submission number 2022-152-IMP-129, date of approval 12 January 2023 by the Institute Ethics Committee, SGPGIMS, Lucknow).^10–15^ All the included patients fulfilled either the 1990 ACR or the 2022 ACR-EULAR classification criteria.^16,17^ Data regarding their clinical characteristics and angiographic subtype (Hata’s) were tabulated.^18^ Their disease activity as per physician global assessment (PGA, active or inactive), Indian TAK Clinical Disease Activity Score (ITAS2010), Disease Extent Index in TAK (DEI.TAK), acute phase reactants (erythrocyte sedimentation rate and C-reactive protein), and damage using the Large Vessel Vasculitis Index of Damage (LVVID) at the time of assessment of PROM were recorded.^19–21^ Details of treatment with glucocorticoids and disease-modifying anti-rheumatic drugs (DMARDs) and the initiation of medications for anxiety or depression were noted.

For all these patients with TAK, a comprehensive assessment of PROMs was undertaken, i.e., quality of life using the EuroQol-5D-3L [EQ-5D, individual components and visual analog scale (VAS)],^22^ disability using the Health Assessment Questionnaire (HAQ,^23^ physical activity – Metabolic Equivalents of Task (METs) using the International Physical Activity Questionnaire – Short Form (IPAQ-SF),^24^ work impairment using the Work Productivity and Activity Impairment – General Health (WPAI-GH),^25^ anxiety using the Generalised Anxiety Disorder seven items (GAD-7) scale,^26^ depression using the Patient Health Questionnaire nine items (PHQ-9) scale,^27^ fatigue using the Multidisciplinary Fatigue Inventory (MFI) and Functional Assessment of Chronic Illness Therapy (FACIT) and fibromyalgia using the 2016 ACR criteria for fibromyalgia.^28–30^ Higher MFI scores and lower FACIT scores indicate greater fatigue.^28,29^ The severity of disability (HAQ ≥1 suggested significant disability), anxiety (minimal 0–4, mild 5–9, moderate 10–14, severe 15–21 on the GAD-7 scale) and depression (minimal 0–4, mild 5–9, moderate 10–14, moderately severe 15–19, severe 20–27 on the PHQ-9 scale) were graded.^8,26,27^ All the questionnaires except the IPAQ-SF and MFI were self-administered by patients in their validated translations in the local vernacular (Hindi). The English versions of the IPAQ-SF and MFI were administered by an investigator to the patients who also assessed the fulfilment of the 2016 revised ACR criteria for fibromyalgia and assessed the polysymptomatic distress score (PSD, a summation of the scores obtained in the widespread pain index and symptom severity scales).^30^ Licenses were obtained from the copyright holders to use the EuroQol-5D-3L, HAQ, MFI and FACIT. The rest of the questionnaires were available for free use online. A repeat assessment of all the PROMs was done at least 4 months after the initial assessment to assess the stability of the observations. For comparison, age and sex-similar healthy control subjects without known comorbidities among the staff members of the institution were recruited for comparison.

Continuous variables were presented using means [± standard deviation(SD)] and compared using unpaired or paired Student’s t-test as appropriate. Categorical variables were presented as numbers with percentages and compared using Fisher’s exact test. The primary analyses compared PROMs between patients with TAK and healthy controls using unpaired analyses and the stability of PROMs over time in patients with TAK using paired analyses. Secondary analyses compared PROMs between patients with TAK with active or inactive disease by PGA using unpaired analyses. PROMs were correlated with DEI.TAK, ITAS2010 and LVVID scores using Pearson’s correlation coefficient. Linear regression was used to adjust for any differences in PROMs between patients with TAK with active or inactive disease (by PGA) for LVVID scores. Further exploratory analyses were directed towards predictors of PROMs in patients with TAK. To explore the association of PROMs with disease features, the PROMs were compared based on sex (female vs male), clinical and angiographic features. Those patients who had been initiated on medications for anxiety and depression were excluded from the serial assessment of GAD-7 and PHQ-9. For the remaining patients, the evolution of the severity of anxiety and depression scores was visualised using alluvial plots made using RAWGraphs.^31^ Spider charts generated using Microsoft Excel for Mac (version 16.78.3) were used to depict the differences in the mean scores of individual components of Euro-Qol-5D (mobility, self-care, usual activities, pain/discomfort, anxiety/depression) between patients with TAK and healthy controls, patients with TAK with active or inactive disease, and on serial follow-up. Statistical significance was inferred if the p-value was <0.05. All the other analyses were performed using Prism 10 for macOS (GraphPad Software, LLC, USA) or STATA 18 B/E (StataCorp, USA). Given the rarity of TAK and the exploratory design of the study, a formal sample size calculation was not undertaken for this study.

## RESULTS

### Characteristics of the included patients

Eighty-four patients with TAK (mean±SD age 35.61±11.95 years, 61 females, disease duration 95.27 ± 85.41 months) and sixty-one healthy controls of comparable age (33.62±9.91 years, unpaired Student’s t test p-value = 0.278) and sex (45 females, Fisher’s exact p value>0.999) were recruited. Thirteen patients with TAK had active disease by PGA. Eighty-two patients with TAK had recordable damage on the LVVID (mean ± SD 3.42 ± 1.99). Hypertension, vascular bruits and pulse loss were the most prevalent clinical features. Hata’s angiographic subtype V was the most prevalent (61.9%). About two-thirds of patients were on glucocorticoids (54/84) or DMARDs (58/84, mostly conventional DMARDs). A repeat assessment of the PROMs was performed for 75 patients at an interval of 7.53 ± 2.42 months). The serial assessment of anxiety and depression was performed in 63 patients as twelve patients had been initiated on antidepressants. The characteristics of the cohort are summarised in **Table 1**.

**Table 1.** Characteristics of the patients with TAK (n=84).

**Parameter**	
Age at enrolment (years) (Mean ± SD)	35.61 ± 11.95
Female: Male	61:23
Fulfilled 1990 ACR classification criteria	78/84 (92.86%)
Fulfilled 2022 ACR-EULAR classification criteria	83/84 (98.81%)
Active disease at baseline by physician global assessment [n (%)]	13/84 (15.66%)
Baseline ITAS2010 (Mean ± SD)	1.56 ± 3.67
Baseline DEI.TAK (Mean ± SD)	1.39 ± 3.36
Baseline LVVID (Mean ± SD)	3.42 ± 1.99
Baseline ESR (mm/hour) (Mean ± SD)	34.43 ± 22.04
Baseline CRP (mm/hour) (Mean ± SD)	7.75 ± 10.87

**Clinical features at presentation [n (%)]**	

Constitutional features	37/84 (44.05%)
Neurological features	28/84 (33.33%)
Pulse loss	56/84 (66.66%)
Vascular bruits	61/84 (72.62%)
Upper limb claudication	26/84 (30.96%)
Lower limb claudication	17/84 (20.24%)
Hypertension	65/84 (77.38%)
Aortic regurgitation	4/84 (4.76%)
Impaired renal functions	8/84 (9.52%)
Heart failure	5/84 (5.95%)

**Angiographic subtype [n (%)]**	

I	12/84 (14.29%)
IIA	3/84 (3.57%)
IIB	12/84 (14.29%)
III	1/84 (1.19%)
IV	4/84 (4.76%)
V	52/84 (61.90%)
Pulmonary artery involvement	6/84 (7.14%)
Coronary artery involvement	7/84 (8.33%)

**Immunosuppressive treatment [n (%)]**	

Glucocorticoids	54/84 (64.29%)
Mean starting dose of prednisolone	12.17 ± 12.34 mg
On any DMARD	58/84 (69.05%)
Methotrexate	19/84 (22.62%)
Azathioprine	5/84 (5.95%)
Tacrolimus	32/84 (38.10%)
Mycophenolate	11/84 (13.10%)
Adalimumab	1/84 (1.19%)

Initiated on antidepressant medications*	12/84 (14.28%)

* Sertraline: (n=10), dosulepin (n=1), duloxetine (n=1).

ACR: American College of Rheumatology; CRP: C-reactive protein; DEI.TAK: Disease Extent Index in Takayasu arteritis; DMARD: Disease-modifying anti-rheumatic drugs; ESR: Erythrocyte sedimentation rate; EULAR: European Alliance of Associations for Rheumatology; ITAS2010: Indian Takayasu arteritis clinical disease activity score 2010; SD: Standard deviation; LVVID: Large vessel vasculitis index of damage.

### Primary analyses: Comparison of PROMs between patients with TAK and healthy controls

**Figure 1.**
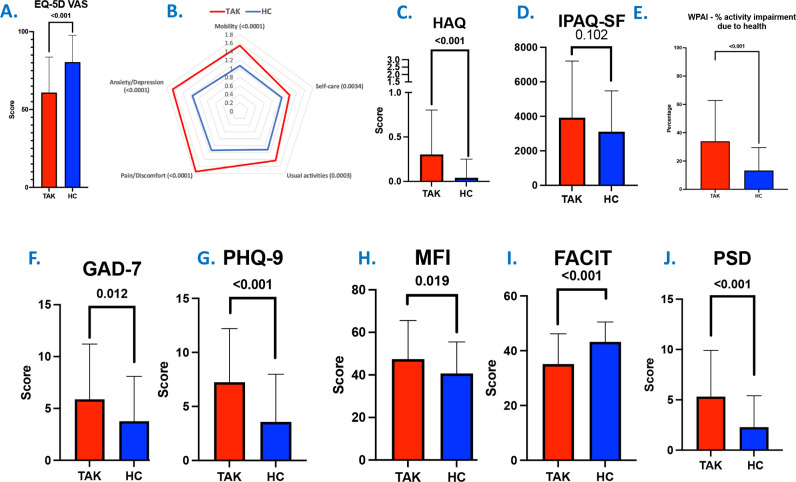
Comparison of PROMs between patients with Takayasu arteritis and healthy controls. Eighty-four patients with TAK were compared with 61 healthy controls. A. EQ-5D VAS B. individual components of the EQ-5D C. HAQ D. IPAQ-SF E. Percentage of work impairment due to health using the WPAI F. GAD-7 G. PHQ-9 H. MFI I. FACIT J. PSD from the 2016 revision of the ACR criteria for fibromyalgia.

Patients with TAK had worse quality of life overall (EQ-5D VAS, **Figure 1A**) as well as in all the individual components of the EQ-5D-3L scale (**Figure 1B**) and disability (HAQ scores, **Figure 1C**) than healthy controls. Similar physical activity was observed in patients with TAK and control subjects (**Figure 1D**). The percentage of activity impairment due to health on the WPAI (**Figure 1E**), anxiety (**Figure 1F**), depression (**Figure 1G**), and fatigue (MFI, **Figure 1H**; FACIT, **Figure 1I**) were all worse in patients with TAK. The prevalence of fibromyalgia was similar between patients with TAK (6/84) and controls (1/61, Fisher’s exact p-value = 0.239), however, PSD was higher in patients with TAK (**Figure 1J**). The prevalence of significant disability (HAQ ≥1) was similar in patients with TAK (7/84) or controls (1/61, Fisher’s exact p-value = 0.139). About one-half of patients with TAK had minimal anxiety whereas a quarter had mild anxiety, and 71% of healthy controls had minimal anxiety assessed on the GAD-7. A third of patients with TAK (versus 71% of healthy controls) had no depression assessed using the PHQ-9. One-half of patients with TAK had mild or moderate depression (versus a quarter of healthy individuals) (**Table 2**).

**Table 2.** Severity of anxiety and depression compared between patients with TAK and healthy controls.

	**TAK (n=84)**	**Healthy controls (n=61)**
**GAD-7**		
Minimal anxiety (0–4)	41 (48.81%)	43 (70.49%)
Mild anxiety (5–9)	23 (27.38%)	11 (18.03%)
Moderate anxiety (10–14)	11 (13.10%)	5 (8.19%)
Severe anxiety (15–21)	9 (10.71%)	2 (3.28%)
**PHQ-9**		
No depression (0–4)	30 (35.71%)	43 (70.49%)
Mild depression (5–9)	26 (30.95%)	11 (18.03%)
Moderate depression (10–14)	20 (23.81%)	4 (6.56%)
Moderately severe depression (15–19)	7 (8.33%)	3 (4.92%)
Severe depression (20–27)	1 (1.2%)	0 (0%)

GAD-7: Generalised Anxiety Disorder assessment 7 questionnaire;

PHQ-9: Patient Health Questionnaire 9.

### Primary analyses: Serial changes in PROMs in patients with TAK

**Figure 2.**
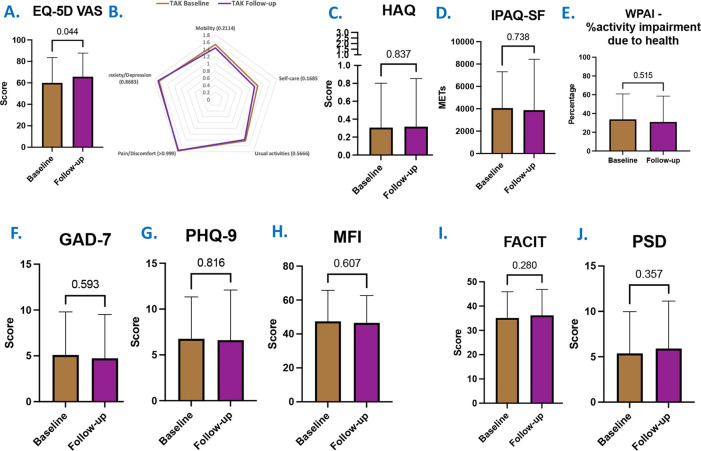
Comparison of PROMs between patients with Takayasu arteritis at baseline or follow-up visits. Serial assessment pf PROMs was undertaken for 75 patients with TAK (63 for GAD-7 and PHQ-9). A. EQ-5D VAS B. individual components of the EQ-5D C. HAQ D. IPAQ-SF E. Percentage of work impairment due to health using the WPAI F. GAD-7 G. PHQ-9 H. MFI I. FACIT J. PSD from the 2016 revision of the ACR criteria for fibromyalgia.

**Figure 3.**
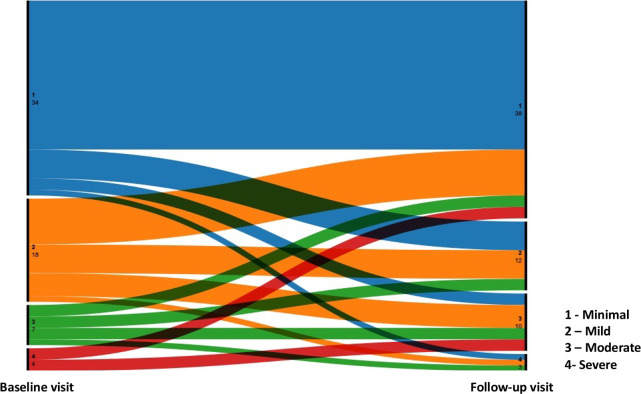
Evolution of the severity of anxiety assessed using the GAD-7 over time in patients with Takayasu arteritis (n=63).

**Figure 4.(above).**
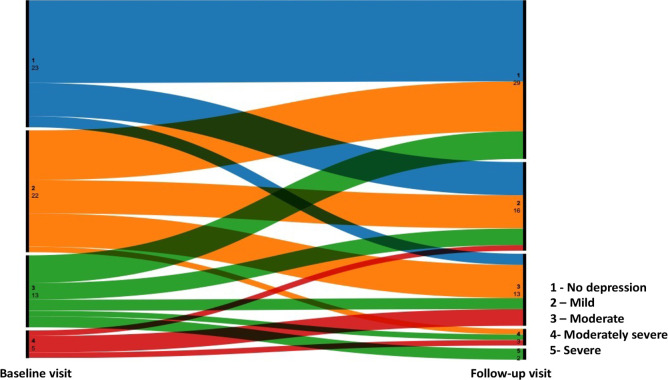
Evolution of the severity of depression assessed using the PHQ-9 over time in patients with Takayasu arteritis (n=63).

Serial assessment of PROMs was available for 75 patients. Serial assessments for anxiety and depression were reported for 63 patients as the remaining patients had been initiated on antidepressant medications after the baseline assessment (**Table 1**). The overall quality of life (**Figure 2A**), individual components of the EQ-5D-3L (**Figure 2B**), disability (**Figure 2C**), physical activity (**Figure 2D**), percentage of activity impairment due to health (**Figure 2E**), anxiety (**Figure 2F**), depression (**Figure 2G**), fatigue (**Figures 2H and 2I**), and PSD scores (**Figure 2J**) were similar on follow-up assessments to that at the initial visit. Similar proportions of patients with TAK fulfilled the 2016 ACR criteria for fibromyalgia at baseline (5/75) or follow-up (7/75) visits (Fisher’s exact p-value = 0.765). The severity of disability (HAQ ≥1, 6/75 at baseline vs 8/75 on follow-up, Fisher’s exact p-value = 0.780), anxiety (**Figure 3**), and depression (**Figure 4**) remained similar over time.

### Secondary analyses: Association between PROMs and disease activity or damage in patients with TAK

A weak negative correlation was observed between ITAS2010 and DEITAK and physical activity (METs) and FACIT scores, whereas a weak positive correlation was observed with PSD scores in patients with TAK. Higher LVVID scores were weakly correlated with worse GAD-7 and PHQ-9 scores (**Table 3**).

**Table 3.** Correlation of patient-reported outcome measures with disease activity and damage scores.

**Parameter**		**EQ-5D VAS**	**HAQ**	**IPAQ-SF METs**	**WPAI (percentage of activity impairment due to health)**	**GAD-7**	**PHQ-9**	**MFI**	**FACIT**	**PSD**
**LVVID**	**r**	−0.02	0.08	−0.01	0.17	0.23	0.30	0.09	−0.12	0.03
	**p**	0.86	0.45	0.90	0.13	**0.03**	**0.01**	0.42	0.26	0.80
**ITAS2010**	**r**	−0.08	0.02	**−0.29**	0.19	0.06	0.21	0.22	**−0.23**	**0.24**
	**p**	0.45	0.86	**0.01**	0.09	0.59	0.06	0.05	**0.03**	**0.03**
**DEI.TAK**	**r**	−0.06	0.02	**−0.29**	0.20	0.06	0.22	0.21	**−0.23**	**0.23**
	**p**	0.58	0.85	**0.01**	0.07	0.60	0.05	0.06	**0.04**	0.04

DEI.TAK: Disease Extent Index in Takayasu arteritis; EQ-5D VAS: EuroQOL 5-dimensions visual analog scale; FACIT: 13-item functional assessment of chronic illness fatigue scale; GAD-7: Generalised Anxiety Disorder assessment 7 questionnaire; HAQ: Healthy Assessment Questionnaire; IPAQ-SF: International Physical Activity Questionnaire: Short Form; ITAS2010: Indian Takayasu arteritis clinical disease activity score 2010; LVVID: Large vessel vasculitis index of damage; PHQ-9: Patient Health Questionnaire 9; PSD: Polysymptomatic Distress Scale; MFI: Multidisciplinary Fatigue Inventory; WPAI: Work Productivity and Activity Index (percentage of activity impairment due to health).

**Figure 5.(below).**
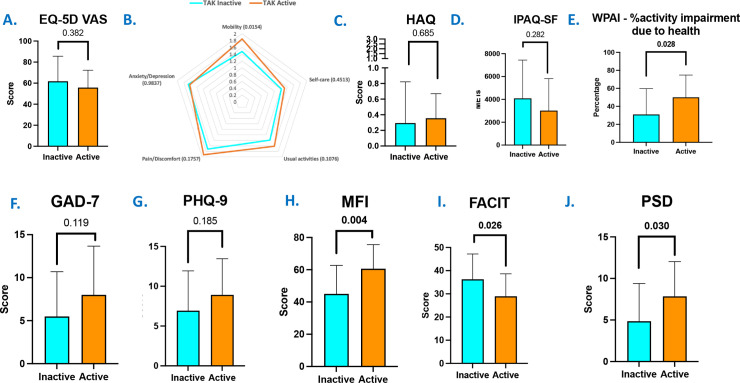
Comparison of PROMs between patients with Takayasu arteritis with active or inactive disease as per physician global assessment. Thirteen patients with active TAK were compared with 71 with inactive disease. A. EQ-5D VAS B. individual components of the EQ-5D C. HAQ D. IPAQ-SF E. Percentage of work impairment due to health using the WPAI F. GAD-7 G. PHQ-9 H. MFI I. FACIT J. PSD from the 2016 revision of the ACR criteria for fibromyalgia.

Patients with active TAK had similar overall quality of life (EQ-5D VAS, **Figure 5A**) as well as in most of the individual components of the EQ-5D-3L scale (**Figure 5B**) and similar disability (**Figure 5C)** or physical activity (**Figure 5D)** when compared with those with inactive TAK. The percentage of activity impairment due to health on the WPAI (**Figure 5E**) was worse for patients with active TAK, however, anxiety (**Figure 5F**) and depression (**Figure 5G**) scores were similar. Patients with active TAK had worse fatigue (higher MFI scores, **Figure 5H**, and lower FACIT scores, **Figure 5I**). Similar proportions of patients with active (2/13) or inactive TAK (4/71, Fisher’s exact p-value = 0.231) had fibromyalgia, however, PSD scores were higher for patients with active TAK (**Figure 5J**). After adjustment for damage scores, patients with active TAK had worse activity impairment due to health on the WPAI, fatigue and PSD scores than those with inactive TAK (**Table 4**). The prevalence of significant disability (HAQ ≥1) was similar in patients with active (0/13) or inactive TAK (7/71, Fisher’s exact p-value = 0.589).

**Table 4.** Linear regression models for patient-reported outcome measures in patients with TAK with active or inactive disease adjusted for LVVID scores.

**PROM**	**Unadjusted Coefficient (with 95%CI)**	**Adjusted Coefficient (with 95%CI)**	**p value**
EQ-5D VAS	−6.06 (−19.77, 7.65)	−6.00 (−19.84, 7.84)	0.391
HAQ	0.06 (−0.24, 0.36)	0.05 (−0.25, 0.36)	0.727
IPAQ-SF	−1071.67 (−3040.57, 897.23)	−1067.86 (−3055.00, 919.29)	0.288
WPAI	19.01 (2.09, 35.94)	18.10 (1.22, 34.98)	0.036
GAD-7	2.51 (−0.66, 5.67)	2.26 (−0.86, 5.37)	0.153
PHQ-9	1.99 (−0.97, 4.96)	1.69 (−1.17, 4.55)	0.243
MFI	15.60 (5.20, 26.01)	15.35 (4.88, 25.83)	0.005
FACIT	−7.36 (13.81, −0.91)	−7.12 (−13.59, −0.64)	0.032
PSD	2.99 (0.30, 5.68)	2.98 (0.26, 5.69)	0.032

Damage was assessed using the LVVID (large vessel vasculitis index of damage).

95%CI: 95% confidence intervals; EQ-5D VAS: EuroQOL 5-dimensions visual analog scale; FACIT: 13-item functional assessment of chronic illness fatigue scale; GAD-7: Generalised Anxiety Disorder assessment 7 questionnaire; HAQ: Healthy Assessment Questionnaire; IPAQ-SF: International Physical Activity Questionnaire: Short Form; LVVID: Large Vessel Vasculitis Index of Damage; MFI: Multidisciplinary Fatigue Inventory; PHQ-9: Patient Health Questionnaire 9; PSD: Polysymptomatic Distress Scale; PROM: Patient-reported outcome measure; WPAI: Work Productivity and Activity Index (percentage of activity impairment due to health).

### Exploratory analyses: Association between PROMs and demographic, clinical and angiographic features of patients with TAK

PSD scores were higher for female patients with TAK when compared with males, however, the proportion of patients with fibromyalgia and the other assessed PROMs were similar (**Table 5**).

**Table 5.** Comparison of PROMs in female or male patients with TAK.

**PROM**	**Females (n=61)**	**Males (n=23)**	p value*
EQ-5D VAS	60.41 ± 21.92	62.17 ± 25.53	0.754
HAQ	0.28 ± 0.48	0.36 ± 0.56	0.538
IPAQ-SF METs	4038.66 ± 3578.54	3608.28 ± 2372.12	0.595
WPAI (%)	36.89 ± 29.24	26.09 ± 26.92	0.127
GAD-7	6.44 ± 5.52	4.39 ± 4.55	0.116
PHQ-9	7.80 ± 4.91	5.74 ± 4.91	0.089
MFI	49.20 ± 18.83	42.74 ± 15.60	0.147
FACIT	34.34 ± 11.31	37.26 ± 10.11	0.282
PSD	6.20 ± 4.80	3.00 ± 2.97	**0.004**

Fibromyalgia	6/61	0/23	0.119

* Unpaired Student’s t-test for means with standard deviations, Fisher’s exact test for proportions.

EQ-5D VAS: EuroQOL 5-dimensions visual analog scale; FACIT: 13-item functional assessment of chronic illness fatigue scale; GAD-7: Generalised Anxiety Disorder assessment 7 questionnaire; HAQ: Healthy Assessment Questionnaire; IPAQ-SF: International Physical Activity Questionnaire: Short Form; LVVID: Large Vessel Vasculitis Index of Damage; MFI: Multidisciplinary Fatigue Inventory; PHQ-9: Patient Health Questionnaire 9; PSD: Polysymptomatic Distress Scale; PROM: Patient-reported outcome measure; WPAI: Work Productivity and Activity Index (percentage of activity impairment due to health).

Patients with TAK with upper limb claudication had significantly worse overall quality of life (EQ-5D VAS), higher anxiety and depression, fatigue (FACIT) and PSD scores and higher physical activity than those without. Those with lower limb claudication, heart failure or vascular bruits had significantly worse depression scores, however, the other PROMs were similar when compared with those without. The presence of hypertension was associated with significantly better fatigue (MFI) and PSD scores but the other PROMs were similar to those without hypertension (**Table 6**).

**Table 6.** Comparison of PROMs in patients with TAK based on disease phenotype.

**PROM**			p value*
**Constitutional feature**	**Yes (n=37)**	**No (n=47)**	

EQ-5D VAS	61.76 ± 21.09	60.21 ± 24.29	0.760
HAQ	0.25 ± 0.47	0.34 ± 0.52	0.417
IPAQ-SF	4489.65 ± 3179.73	3473.01 ± 3329.26	0.160
WPAI (%)	38.65 ± 28.10	30.21 ± 29.23	0.185
GAD-7	6.35 ± 5.30	5.51 ± 5.37	0.476
PHQ-9	7.32 ± 4.47	7.17 ± 5.37	0.889
MFI	49.14 ± 19.38	46.09 ± 17.20	0.448
FACIT	34.27 ± 10.31	35.83 ± 11.60	0.523
PSD	5.70 ± 4.23	5.02 ± 4.88	0.503

Fibromyalgia	2/37	4/47	0.690

**Neurological features**	**Yes (n=28)**	**No (n=56)**	

EQ-5D VAS	61.61 ± 19.25	60.54 ± 24.56	0.841

HAQ	0.44 ± 0.68	0.23 ± 0.37	0.073

IPAQ-SF	3810.54 ± 3576.94	3975.96 ± 3160.21	0.829

WPAI (%)	31.07 ± 30.59	35.36 ± 28.15	0.525

GAD-7	6.00 ± 5.67	5.82 ± 5.19	0.886

PHQ-9	7.11 ± 5.17	7.30 ± 4.90	0.866

MFI	47.36 ± 20.73	47.46 ± 16.91	0.980

FACIT	34.68 ± 12.10	35.38 ± 10.53	0.787

PSD	4.86 ± 4.06	5.55 ± 4.85	0.515

Fibromyalgia	1/28	5/56	0.658

**Pulse loss**	**Yes (n=56)**	**No (n=28)**	

EQ-5D VAS	59.91 ± 22.29	62.86 ± 24.13	0.580

HAQ	0.29 ± 0.42	0.34 ± 0.63	0.646

IPAQ-SF	4077.55 ± 3567.02	3607.34 ± 2661.92	0.539

WPAI (%)	36.07 ± 27.35	29.64 ± 31.80	0.339

GAD-7	6.00 ± 5.66	5.64 ± 4.66	0.774

PHQ-9	7.38 ± 5.08	6.96 ± 4.80	0.723

MFI	46.75 ± 17.25	48.79 ± 20.08	0.631

FACIT	35.00 ± 11.19	35.43 ± 10.83	0.868

PSD	5.45 ± 4.66	5.07 ± 4.53	0.726

Fibromyalgia	4/56	2/28	>0.999

**Vascular bruits**	**Yes (n=61)**	**No (n=23)**	

EQ-5D VAS	62.54 ± 22.83	56.52 ± 22.69	0.284

HAQ	0.35 ± 0.56	0.17 ± 0.27	0.145

IPAQ-SF	3737.63 ± 3358.38	4406.65 ± 3097.25	0.408

WPAI (%)	34.59 ± 30.31	32.17 ± 25.22	0.735

GAD-7	6.13 ± 5.25	5.22 ± 5.59	0.486

PHQ-9	7.93 ± 5.26	5.39 ± 3.54	**0.036**

MFI	47.67 ± 18.03	46.78 ± 18.83	0.843

FACIT	33.93 ± 11.32	38.35 ± 9.66	0.102

PSD	5.52 ± 5.00	4.78 ± 3.30	0.512

Fibromyalgia	6/61	0/23	0.182

**Upper limb claudication**	**Yes (n=26)**	**No (n=58)**	

EQ-5D VAS	50.38 ± 22.76	65.60 ± 21.40	**0.004**

HAQ	0.33 ± 0.34	0.29 ± 0.56	0.732

IPAQ-SF	5082.75 ± 3697.47	3399.95 ± 2969.51	**0.029**

WPAI (%)	41.54 ± 25.56	30.52 ± 29.82	0.106

GAD-7	7.65 ± 5.63	5.09 ± 5.02	**0.040**

PHQ-9	9.12 ± 4.67	6.40 ± 4.90	**0.019**

MFI	52.85 ± 18.29	45.00 ± 17.70	0.067

FACIT	31.35 ± 10.87	36.84 ± 10.73	**0.034**

PSD	7.65 ± 5.28	4.28 ± 3.86	**0.001**

Fibromyalgia	3/26	3/58	0.295

**Lower limb claudication**	**Yes (n=17)**	**No (n=67)**	

EQ-5D VAS	54.71 ± 28.69	62.46 ± 21.04	0.213

HAQ	0.33 ± 0.47	0.30 ± 0.51	0.803

IPAQ-SF	4221.82 ± 2987.39	3844.44 ± 3372.17	0.675

WPAI (%)	45.88 ± 30.63	30.90 ± 27.84	0.056

GAD-7	7.82 ± 5.36	5.39 ± 5.24	0.092

PHQ-9	10.29 ± 5.97	6.46 ± 4.40	**0.004**

MFI	48.35 ± 17.74	47.19 ± 18.37	0.816

FACIT	34.06 ± 10.46	35.42 ± 11.21	0.652

PSD	5.71 ± 3.74	5.22 ± 4.80	0.701

Fibromyalgia	1/17	5/67	>0.999

**Hypertension**	**Yes (n=65)**	**No (n=19)**	

EQ-5D VAS	61.85 ± 23.86	57.63 ± 19.03	0.482

HAQ	0.29 ± 0.54	0.34 ± 0.35	0.705

IPAQ-SF	3706.29 ± 3175.49	4654.74 ± 3625.99	0.271

WPAI (%)	30.93 ± 28.32	44.21 ± 29.12	0.078

GAD-7	5.52 ± 5.18	7.11 ± 5.76	0.257

PHQ-9	6.80 ± 5.01	8.74 ± 4.62	0.136

MFI	44.65 ± 16.44	56.95 ± 20.81	**0.009**

FACIT	35.94 ± 11.28	32.42 ± 9.82	0.223

PSD	4.74 ± 4.51	7.32 ± 4.41	**0.030**

Fibromyalgia	5/65	1/19	>0.999

**Aortic regurgitation**	**Yes (n=4)**	**No (n=80)**	

EQ-5D VAS	65.00 ± 33.17	60.69 ± 22.46	0.715

HAQ	0.31 ± 0.33	0.30 ± 0.51	0.971

IPAQ-SF	4421.25 ± 2571.80	3895.79 ± 3326.82	0.757

WPAI (%)	37.50 ± 23.63	33.75 ± 29.23	0.802

GAD-7	6.25 ± 1.71	5.86 ± 5.45	0.888

PHQ-9	9.50 ± 3.87	7.13 ± 5.00	0.354

MFI	51.75 ± 8.96	47.21 ± 18.49	0.628

FACIT	30.00 ± 8.12	35.40 ± 11.11	0.342

PSD	5.75 ± 5.06	5.30 ± 4.60	0.850

Fibromyalgia	0/4	6/80	>0.999

**Impaired renal functions**	**Yes (n=8)**	**No (n=76)**	

EQ-5D VAS	55.63 ± 27.18	61.45 ±22.45	0.496

HAQ	0.38 ± 0.64	0.30 ± 0.49	0.674

IPAQ-SF	3189.00 ± 3037.55	3997.85 ± 3318.48	0.511

WPAI (%)	43.75 ± 38.89	32.89 ± 27.75	0.315

GAD-7	8.50 ± 6.02	5.61 ± 5.21	0.145

PHQ-9	10.00 ± 6.97	6.95 ± 4.67	0.098

MFI	46.38 ± 13.57	47.54 ± 18.63	0.864

FACIT	32.75 ± 15.64	35.39 ± 10.52	0.521

PSD	4.75 ± 5.47	5.38 ± 4.53	0.714

Fibromyalgia	2/8	4/76	0.100

Heart failure	Yes (n=5)	No (n=79)	

EQ-5D VAS	64.00 ± 29.45	60.70 ± 22.56	0.756

HAQ	0.30 ± 0.31	0.30 ± 0.51	0.987

IPAQ-SF	4318.80 ± 2934.15	3895.63 ± 3320.42	0.782

WPAI (%)	54.00 ± 37.82	32.66 ± 28.04	0.109

GAD-7	6.20 ± 3.42	5.86 ± 5.44	0.891

PHQ-9	12.40 ± 4.62	6.91 ± 4.82	**0.016**

MFI	52.20 ± 21.46	47.13 ± 18.03	0.548

FACIT	30.80 ± 9.60	35.42 ± 11.09	0.366

PSD	4.40 ± 3.65	5.38 ± 4.66	0.646

Fibromyalgia	0/5	6/79	>0.999

* Unpaired Student’s t-test for means with standard deviations, Fisher’s exact test for proportions.

EQ-5D VAS: EuroQOL 5-dimensions visual analog scale; FACIT: 13-item functional assessment of chronic illness fatigue scale; GAD-7: Generalised Anxiety Disorder assessment 7 questionnaire; HAQ: Healthy Assessment Questionnaire; IPAQ-SF: International Physical Activity Questionnaire: Short Form; LVVID: Large Vessel Vasculitis Index of Damage; MFI: Multidisciplinary Fatigue Inventory; PHQ-9: Patient Health Questionnaire 9; PSD: Polysymptomatic Distress Scale; PROM: Patient-reported outcome measure; WPAI: Work Productivity and Activity Index (percentage of activity impairment due to health).

Since Hata’s angiographic subtype V was the most prevalent (61.9%), we compared PROMs between patients with Hata’s type V and other angiographic subtypes. However, no significant differences were observed (**Table 7**).

**Table 7.** Comparison of PROMs in patients with TAK based on angiographic subtype.

**PROM**	**Hata’s Type V (n=52)**	**non-Hata’s Type V (n=32)**	p value*
EQ-5D VAS	62.79 ± 23.48	57.82 ± 21.70	0.335
HAQ	0.26 ± 0.42	0.38 ± 0.61	0.307
IPAQ-SF	3864.85 ± 3449.31	4011.77 ± 3048.18	0.844
WPAI	30.58 ± 25.16	39.38 ± 33.79	0.177
GAD-7	5.79 ± 5.04	6.03 ± 5.84	0.841
PHQ-9	6.77 ± 4.87	8.00 ± 5.10	0.272
MFI	45.85 ± 16.71	50.00 ± 20.28	0.311
FACIT	35.92 ± 10.99	33.88 ± 11.10	0.411
PSD	4.88 ± 4.25	6.03 ± 5.08	0.269
Fibromyalgia	4/52	2/32	p>0.999

* Unpaired Student’s t-test for means with standard deviations, Fisher’s exact test for proportions. EQ-5D VAS: EuroQOL 5-dimensions visual analog scale; FACIT: 13-item functional assessment of chronic illness fatigue scale; GAD-7: Generalised Anxiety Disorder assessment 7 questionnaire; HAQ: Healthy Assessment Questionnaire; IPAQ-SF: International Physical Activity Questionnaire: Short Form; LVVID: Large Vessel Vasculitis Index of Damage; MFI: Multidisciplinary Fatigue Inventory; PHQ-9: Patient Health Questionnaire 9; PSD: Polysymptomatic Distress Scale; PROM: Patient-reported outcome measure; WPAI: Work Productivity and Activity Index (percentage of activity impairment due to health).

## DISCUSSION

To the best of our knowledge, this is the first comprehensive assessment of PROMs in patients with TAK. Patients with TAK had worse quality of life, disability, activity impairment due to health, anxiety, depression and fatigue than age and sex-similar healthy controls. The various PROMs assessed in the patients with TAK remained stable on follow-up. Secondary analyses revealed that worse physical activity, fatigue and PSD scores were associated with the TAK disease activity measures ITAS2010 and DEI.TAK. Anxiety and depression were correlated with damage scores. After adjustment for damage scores, patients with active TAK had worse impairment of activity due to health, fatigue and PSD scores. Exploratory analyses revealed overall worse PROMs in patients with TAK with upper limb claudication and worse depression in those with lower limb claudication, heart failure, or vascular bruits.

We observed worse quality of life (QoL) scores overall as well as in the individual domains of the EQ-5D-3L scale in patients with TAK than in healthy controls. Comparable impairments in QoL have been reported in patients with TAK and other rheumatic diseases. Akar et al reported a similar degree of perturbation in QoL assessed using the SF36 in patients with TAK, rheumatoid arthritis or ankylosing spondylitis.^32^ Rimland and colleagues reported similar QoL using the SF36 in patients with TAK or Giant Cell Arteritis.^33^ Similar to that already known, patients with TAK in our cohort had worse disability, anxiety and depression, and fatigue than healthy controls.^8^ The use of two scales to assess fatigue (where higher MFI scores and lower FACIT scores indicate more fatigue) and the consistency of observations with these two scales lends confidence to our observation of worse fatigue in patients with TAK. A recent study of 328 patients (13 with TAK) with vasculitis from North America reported frailty or pre-frailty in 63% patients. The presence of frailty was associated with depression and fatigue.^34^ However, we have not assessed frailty in our patients with TAK, this merits further exploration.

Unlike our observation of similar physical activity between patients with TAK and control subjects, Dos Santos and colleagues had observed lower physical activity levels in patients with TAK than healthy controls.^35^ Physical activity is an important protective factor for cardiovascular disease whose risk is increased in patients with TAK which also drives a higher mortality rate in such patients.^12,36^ Therefore, patients with TAK should be routinely counselled during clinic visits to undertake regular exercises. Although the absolute scores on the polysymptomatic distress scale were significantly higher for patients with TAK, the prevalence of fibromyalgia was similar in patients with TAK and healthy controls. Alibaz-Oner and colleagues had also noticed that similar proportions of patients with TAK or healthy controls fulfilled the ACR criteria for fibromyalgia.^37^

We observed the stability of reported PROMs over time in patients with TAK. Similar observations have been reported for QoL, disability depression, and anxiety in patients with TAK. Alibaz-Oner and colleagues reported similar SF-36, HAQ and anxiety/depression scores in patients with TAK when assessed six months apart.^37^ Campochiaro and colleagues reported similar HAQ scores in patients with TAK before or after treatment with infliximab biosimilar.^38^ Oliveira and colleagues reported similar HAQ scores in patients with TAK before or after the administration of exercise therapy.^39^ However, in other rheumatic diseases such as axial spondyloarthropathy, an improvement in disability and work impairment have been reported after the initiation of biologic DMARDs.^40^

Clinically assessed damage has been scarcely studied in patients with TAK.^3,41^ Omma et al. reported a weak but significant association between Vasculitis Damage Index (VDI) scores and the physical and mental component scores of the SF-36.^42^ However, the VDI is designed largely for use in small and medium vasculitis.^4^ The LVVID has been recently validated in patients with TAK.^21^ We observed a weak significant association of LVVID with anxiety and depression scores but not with other PROMs in patients with TAK. Therefore, we undertook an adjustment with LVVID scores for the comparison of PROMs between active and inactive TAK. Despite such an adjustment, we observed that patients with TAK with active disease had a greater impairment of physical activity due to health and worse fatigue than those with inactive TAK. A weak correlation was observed between ITAS2010 or DEI.TAK scores and the severity of fatigue. No association between quality of life, anxiety, or depression were observed with active disease in patients with TAK from this study. The literature in this area is conflicting. Akar et al reported inconsistent associations between QoL assessed using the SF-36 and TAK disease activity.^32^ On the contrary, Quartuccio and colleagues reported significant improvements in SF-36 scores upon treatment with infliximab in patients with treatment-refractory TAK.^43^ Similar improvements in SF-36 were reported in a RCT of tocilizumab in TAK.^44^ Erdal et al. reported a greater impairment of physical activity in patients with active TAK when assessed using the WPAI.^45^ Contrary to our observations, they reported worse anxiety and depression scores on the Hospital Anxiety and Depression Scale in patients with active TAK when compared with those with inactive disease.^45^ Zhang and colleagues also noted an association between TAK disease activity scores, active disease, and circulating levels of interleukin-6 with depression.^46,47^ While previous studies have reported worse fatigue in patients with TAK than control subjects, our observation of the association between fatigue and active TAK has not been reported before.^8,48^ In other rheumatic diseases such as rheumatoid arthritis, fatigue has been associated with disease activity.^49^ Contrary to a previous study by Alibaz-Oner and colleagues, we did not identify an association between fibromyalgia and active TAK.^37^ Akin to our observation, Erdal and colleagues noted an impairment of activity in patients with TAK with active than with inactive disease.^45^

Few RCTs are available to guide the management of patients with TAK.^3,50^ Only two of the published RCTs in TAK assessed PROMs. Nakaoka and colleagues reported a sustained improvement in both the physical and mental component scales of the Short Form Health Survey 36 item (SF36) QoL scale from recruitment to the end of the open-label extension period of 96 weeks.^44^ Astley et al. reported better improvements in the physical component of the SF36 in patients with paediatric-onset TAK on supervised home-based exercise regimen when compared with a control group receiving standard of care therapy only.^51^ Wang et al. reported similar improvements in physical and mental component scales of the SF36 in patients with TAK treated with adalimumab or tocilizumab.^52^ The inclusion of a more comprehensive assessment of PROMs is essential for future trials of patients with TAK.

In a study of 50 patients with Behcet’s disease, a variable vessel vasculitis, patients with major or minor organ involvement were observed to have similar impairment of quality of life, sleep, anxiety, and fatigue.^53^ However, the association between specific clinical features of TAK and PROMs has been scarcely reported in the literature. Exploratory analyses in the present study revealed that patients with TAK with upper limb claudication had impairment of multiple PROMs when compared with those without. The presence of lower limb claudication, heart failure or vascular bruits were associated with more severe depression.

The PROMs used in the study were generic since a PROM specific to TAK is yet to be developed. Attempts have been made to understand the perspectives of patients with TAK. A qualitative study involving 31 patients with TAK from Turkey and the United States of America identified pain, discomfort as important concerns of patients with TAK with active or inactive disease, fatigue in those with active TAK, and emotional impact in inactive TAK.^54^ Future studies should be conducted in this area to develop a TAK-specific PROM like those that exist for other rheumatic diseases such as the scleroderma HAQ.^55^

There were limitations to our study. Prevalent rather than newly-diagnosed patients with TAK were included in the study. The inclusion of newly-diagnosed treatment-naïve patients with TAK would inform more clearly how PROMs change following treatment. In this context, few patients included in the study had active disease. Therefore, an assessment of changes in PROMs following improvements in disease activity in patients with TAK was not performed. This is an avenue for further research. Future studies should also evaluate the effect of different treatments (with immunosuppressive medications or surgical interventions) on PROMs in patients with TAK. While many previous studies have used the SF-36 to assess QoL in patients with TAK, we used the EQ-5D-3L due to its accessibility and availability without a fee for academic purposes. A formal sample size calculation was not performed given the exploratory design of the study to comprehensively evaluate the gamut of PROMs in patients with TAK as well as the rarity of TAK.

## CONCLUSION

The findings of the study highlight worse quality of life, disability, anxiety, depression, and fatigue in patients with TAK which remain stable over time. However, the prevalence of fibromyalgia was similar to healthy controls. Active disease was associated with greater impairment of physical activity due to health and fatigue but not the other PROMs. Rheumatologists should routinely assess and address any impairment of PROMs in patients with TAK to improve their overall care. The evaluation of interventions to improve PROMs in patients with TAK forms an important research agenda.

## Data Availability

All the analyses performed for this article have been reported in the main text or the supplementary files. Anonymised data pertaining to the article shall be shared on reasonable request to the corresponding author.

## PERMISSION TO RE-USE QUESTIONNAIRES

Licenses were obtained from the copyright holders to use the EuroQol-5D-3L, HAQ, MFI and FACIT. The rest of the questionnaires were available for free use online.

## SUPPORT FROM EXTERNAL EDITING AGENCIES

There was no support for the manuscript from any editing agencies.

## PRIOR CONFERENCE PRESENTATIONS

Presented as a poster at the 21st International Vasculitis Workshop at Barcelona, Spain in April 2024 (Thakare DR, Rathore U, Singh K, Agarwal V, Misra DP. Patients with Takayasu arteritis experience worse quality of life, physical function, anxiety, depression, and fatigue than healthy individuals which persist over time. doi: 10.5281/zenodo.11068008). Abstract published in the abstract book of EULAR 24 European Congress of Rheumatology at Vienna in June 2024 (Misra DP, Thakare DR, Rathore U, Singh K, Agarwal V. AB1304 PATIENTS WITH TAKAYASU ARTERITIS EXPERIENCE WORSE QUALITY OF LIFE, PHYSICAL FUNCTION, WORK PRODUCTIVITY, ANXIETY, DEPRESSION, AND FATIGUE THAN HEALTHY INDIVIDUALS WHICH PERSIST OVER TIME. Ann Rheum Dis 2024;83:1999. doi: 10.1136/annrheumdis-2024-eular.3730).
